# Outcomes of a Blended Care Coaching Program for Clients Presenting With Moderate Levels of Anxiety and Depression: Pragmatic Retrospective Study

**DOI:** 10.2196/32100

**Published:** 2021-10-21

**Authors:** Monica S Wu, Shih-Yin Chen, Robert E Wickham, Shane O’Neil-Hart, Connie Chen, Anita Lungu

**Affiliations:** 1 Lyra Health Burlingame, CA United States; 2 Department of Psychological Sciences Northern Arizona University Flagstaff, AZ United States; 3 Department of Psychology Palo Alto University Palo Alto, CA United States

**Keywords:** blended care, coaching, cognitive, behavior, depression, anxiety, digital health, retrospective, mental health, CBT, cognitive behavioral therapy, outcome, video conference

## Abstract

**Background:**

Depression and anxiety are leading causes of disability worldwide, but access to quality mental health care is limited by myriad factors. Cognitive-behavioral coaching is rooted in evidence-based principles and has the potential to address some of these unmet care needs. Harnessing technology to facilitate broader dissemination within a blended care model shows additional promise for overcoming barriers to care.

**Objective:**

The aim of this study is to evaluate the outcomes of a blended care coaching (BCC) program for clients presenting with moderate levels of anxiety and depression in real-world settings.

**Methods:**

This study examined retrospective data from US-based individuals (N=1496) who presented with moderate levels of depression and anxiety symptoms and who received blended care coaching services. Using a short-term framework, clients met with coaches via a secure video conference platform and also received digital video lessons and exercises. To evaluate the effectiveness of the BCC program, mixed effects modeling was used to examine growth trajectories of anxiety and depression scores over the course of care.

**Results:**

Out of the total sample of 1496 clients, 75.9% (n=1136) demonstrated reliable improvement, and 88.6% (n=1326) recovered based on either the Generalized Anxiety Disorder-7 scale (anxiety) or Patient Health Questionnaire-9 (depression). On average, clients exhibited a significant decline in anxiety and depression symptoms during the initial weeks of coaching, with a continued decline over subsequent weeks at a lower rate. Engaging in a coaching session was associated with lower anxiety (b=–1.04) and depression (b=–0.79) symptoms in the same week, as well as lower anxiety (b=–0.74) and depression (b=–0.91) symptoms the following week (*P*<.001).

**Conclusions:**

The BCC program demonstrated strong outcomes in decreasing symptomology for clients presenting with moderate levels of anxiety and depression. When clients received coaching sessions, significant decreases in symptoms were observed, reflecting the importance of session attendance. Additionally, the steepest declines in symptoms tended to occur during the initial weeks of coaching, emphasizing the importance of client buy-in and early engagement. Collectively, these findings have implications for addressing unmet mental health care needs in a more accessible, cost-effective manner.

## Introduction

Depression and anxiety are leading causes of disability worldwide and incur significant societal costs [1,2]. The burden is especially apparent in the workplace, as evidenced by decreased productivity, poorer performance, absenteeism, and medical costs [3,4]. There is a robust empirical base supporting the efficacy of cognitive-behavioral therapy (CBT) for anxiety and depression [5], but access to evidence-based care is encumbered by various barriers. In addition, the stigma associated with receiving psychotherapy, cost of seeing out-of-pocket providers, long waitlists, and limited access to quality providers all contribute to delays in seeking and receiving treatment [3,6].

To bridge this gap, coaching has emerged as a contender to address some of these unmet mental health care needs. Historically, coaching has been proposed to facilitate improvements in well-being and performance in personal or professional capacities, using a results-oriented process [7]. As such, coaching has typically targeted individuals who do not meet criteria for clinically significant mental health problems but may present with elevated stressors or difficulties. Coaching is suited to target these broader life and performance challenges, using approaches such as establishing goals, problem-solving, and enhancing self-efficacy. Indeed, compared to psychotherapy, coaching tends to approach care using a life-enhancing model (encouraging interventions that improve the client’s overall well-being vs treating a “disorder”), and it takes a less directive stance to empower clients to devise their own solutions. Additionally, because coaching does not require advanced educational degrees or formal licensing, more coaches may be available to assist individuals, circumventing limitations in availability for licensed therapists and psychologists.

Within this context, it is important to note that less regulation in the training and qualifications of coaches does lend to more variability to the quality of care and theoretical frameworks used in coaching [8,9].Specifically, coaches have identified myriad psychological approaches in their work, including cognitive-behavioral, psychodynamic, solutions-focused, narrative, positive psychology, and mindfulness [10,11]. Because of the significant natural variability in approaches, it is important to consider how coaching can be augmented with rigorous provider selection, cognitive behavioral training, and quality assurance, and delivered within an evidence-based framework similar to those observed in psychotherapy. To address this need, cognitive-behavioral coaching (CBC) could be a promising way to operationalize and strengthen the theoretical principles used by coaches.

Cognitive-behavioral coaching has the potential to be a cost-effective method for adapting CBT principles for use within a coaching context. However, empirical support for CBC effectiveness has been variable, and the studies that do exist widely differ in their methodology [8,12]. In addition, prior studies have primarily focused on subclinical populations experiencing heightened stress or undesired health outcomes [9,13]. The relative dearth of studies examining the utility of CBC among individuals with more moderate levels of symptomology underscores the need to examine the effectiveness of CBC in a sample that generalizes to real-world settings. Additionally, a great deal of opportunity remains to augment the accessibility and delivery of these services within a short-term framework.

To decrease barriers to access, technological advancements have been increasingly effective in facilitating the dissemination of evidence-based care, especially CBT [14]. More specifically, blended care models have garnered growing empirical support, as the pairing of traditional face-to-face interventions with relevant digital activities has implications for greater dissemination, decreased costs, and robust treatment outcomes [15,16]. Although blended care has largely been examined in therapeutic contexts, there is a notable gap in the field for blended care coaching (BCC). In BCC, face-to-face sessions with coaches (in person or via teletherapy) work symbiotically with digital activities to introduce and reinforce key coaching concepts and skills. In general, the digital part of BCC exhibits a range of flexibility; some coaching programs are more regimented, with preset content and digital activities that are assigned to clients to be completed chronologically, while others are more flexible and personalize the assigned content based on what is discussed in the coaching sessions. Preliminary BCC studies have been tested for health outcomes [17,18]; however, these studies notably use psychologists, physicians, and other professionals with advanced degrees as the coaches. Consequently, further studies are needed to examine the effectiveness of BCC programs for psychological symptoms with greater real-world generalizability.

To date, there have been no studies examining CBC outcomes for addressing moderate levels of anxiety and depression using a blended care model. To fill this gap, the present study will examine the effectiveness of a BCC program in a real-world setting, using retrospective data of US-based clients receiving coaching services through Lyra Health. Establishing the effectiveness of BCC would have implications for addressing unmet mental health care needs in a more accessible, cost-effective manner.

## Methods

### Study Design and Procedures

This retrospective study used existing data from Lyra Health, Inc, and Lyra Clinical Associates for delivering BCC services. Lyra Health offers a behavioral health benefit to companies through which employees and dependents have access to CBC within a blended care model. Clients interested in receiving behavioral health benefits completed an initial battery of assessments to establish a baseline level of severity and determine appropriateness for services. Those who were interested in and qualified for coaching services received an average of 4 coaching sessions over 5 weeks. Assessments of depressive and anxiety symptom severity were collected at each session thereafter to track progress. All coaching sessions, assessments, and digital activities were performed through Lyra’s web-based, Health Insurance Portability and Accountability Act (HIPAA)–compliant platform. This pragmatic, retrospective analysis of deidentified data gathered from coaching offered by Lyra Clinical Associates was determined to not be human subjects research by the Western Institutional Review Board.

### Participants and Data Inclusion

Participants included clients who participated in the BCC program between March 21, 2020, and April 8, 2021. They must have scored above the clinical cutoff for either the Patient Health Questionnaire-9 (PHQ-9, score ≥10) or the Generalized Anxiety Disorder-7 scale (GAD-7, score ≥8) on a valid baseline assessment (N=1740). Participants were excluded if their baseline assessment or second assessment were considered invalid (n=22). Clients were excluded from coaching if their initial scores met these criteria: (1) GAD-7 ≥15, (2) PHQ-9 ≥12, or (3) GAD-7=12-14 *and* PHQ-9 ≥10. Other exclusionary criteria included any past psychiatric hospitalization, suicidal/homicidal ideation in the past year, significant traumatic event in the past 6 months, extensive substance use, current violence in a relationship, mandated reporting concerns, or active treatment with a therapist. Other than high baseline scores, clients were most commonly excluded for currently seeing a therapist (13.3%), panic symptoms leading to change of behavior (12.4%), disordered eating concerns (11.5%), or suicidal ideation or self-harm (17.4%). Approximately 43% of clients searching for care were shown coaching, highlighting that the majority of searchers are not shown coaching due to reporting more severe symptomology or meeting exclusionary criteria.

**Figure 1:**
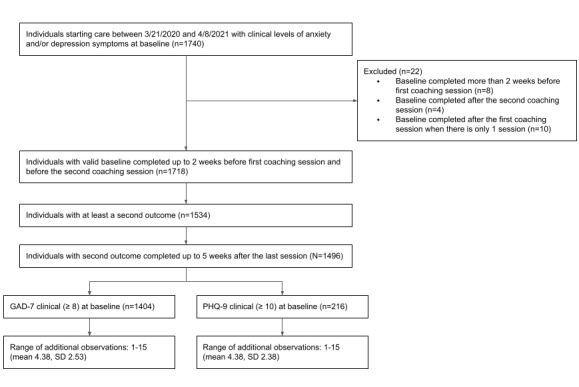
Participant flowchart. GAD-7: Generalized Anxiety Disorder-7 scale; PHQ-9: Patient Health Questionnaire-9.

Coaches had ongoing access to clinical consultation with licensed mental health clinicians for determining when a care transition to therapy was indicated. We also excluded assessments if they were collected more than 12.9 weeks after the first coaching session (representing the mean plus one standard deviation of the coaching duration for the sample). Based on these data inclusion and exclusion criteria, a total of 1496 participants were included in the final sample for analysis (Figure 1). Of the total final sample, there was a 17.5% attrition rate (262/1496 clients), which is defined as the percentage of clients who dropped out of coaching. Demographic information for the final sample is included in Table 1.

**Table 1:** Demographic information and engagement with coaching services. The samples included for the analyses on depression symptoms and on anxiety symptoms partially overlap, as participants with clinical levels of both depression (PHQ-9 score ≥10) and anxiety (GAD-7 score ≥8) were included in both analyses.

Characteristic		Value		
		Entire group (N=1496)	GAD-7^a^ sample (n=1404)	PHQ-9^b^ sample (n=216)
Age, mean (SD)		33.64 (8.62)	33.49 (8.43)	34.28 (9.60)
Female gender, n (%)		921 (61.56)	860 (61.25)	140 (64.81)
**Race/ethnicity**				
	Member of minority group, n (%)	676 (45.19)	631 (44.94)	100 (46.30)
	Unknown, n (%)	68 (4.55)	64 (4.56)	7 (3.24)
Baseline PHQ-9 score, mean (SD)		6.04 (3.00)	5.75 (2.86)	10.74 (1.25)
Baseline GAD-7 score, mean (SD)		9.58 (2.05)	9.85 (1.77)	7.82 (2.54)
Coaching sessions completed, mean (SD)		4.48 (2.31)	4.48 (2.32)	4.50 (2.22)
Duration of care (weeks), mean (SD)		5.73 (4.85)	5.73 (4.84)	5.68 (4.58)

^a^GAD-7: Generalized Anxiety Disorder-7 scale.

^b^PHQ-9: Patient Health Questionnaire-9.

### Coaching Program

The recruited coaches completed an International Coaching Federation–accredited coach training program, demonstrated a minimum of 100 hours of coaching experience, and passed an observational coaching demonstration. Coaches maintained key elements from the coaching approach, including a stance that clients are the experts of their own life, do not need to be fixed, and have the resources to find the solutions to their own problems [19]. Once selected, the coaches underwent intensive, experiential training as part of their onboarding with Lyra, covering evidence-based principles drawn from acceptance and commitment therapy (ACT), dialectical behavior therapy (DBT), and CBT, in addition to an orientation to the blended care content. This training included lectures and demonstrations of core principles (eg, acceptance, values), self-practice/self-reflection of these principles and skills, ongoing consultation and quality assurance, and case presentations. Coaches used a 6-session model (45 minutes each) with the option for clients to request additional sessions; these were generally approved, with the exception of clients needing more intensive care.

### Digital Activities and Coaching Platform

Coaches used Lyra’s secure, web-based platform to see clients via video conference, access assessment data, and assign and review digital activities. Consistent with the blended care model, coaches had access to a variety of digital activities that could be personalized and assigned to clients for use between sessions. Digital activities entailed digital video lessons and exercises, and they were derived from evidence-based interventions (eg, CBT, DBT, ACT). Video lessons introduced core CBT concepts and skills through a storytelling approach, which have been found to be engaging and relatable for users [20]. Digital exercises were akin to digitized versions of traditional CBT worksheets or logs, encouraging practice of skills in between sessions. Examples of concepts and skills covered in these digital activities include mindful awareness, challenging avoidance, cognitive reappraisal, and distress tolerance. Clients could receive feedback on their completed digital exercises through the web-based platform and engage in asynchronous messaging with their coach as needed.

### Self-report Measures

#### Demographics

Demographic information about the client is collected through a self-report questionnaire via the web-based platform, including items assessing sex, race/ethnicity, and birthdate.

#### The PHQ-9

The PHQ-9 is a 9-item self-report questionnaire that assesses depressive symptom severity over the past week [21]. A clinical cutoff score of ≥10 on the PHQ-9 has been validated as a threshold for individuals likely to meet diagnostic criteria for major depression.

#### The GAD-7

The GAD-7 is a 7-item self-report measure that evaluates anxiety symptom severity over the past week [22]. A score of ≥8 on the GAD-7 was used as the most specific and sensitive clinical cutoff for identifying individuals with a diagnosis of generalized anxiety disorder [23].

For both the PHQ-9 and GAD-7, responses are provided on a Likert scale from 0 to 3; a total score can be calculated by summing the items, with higher scores indicating more severe symptomology. Both measures have been used extensively in various clinical trials in different settings, demonstrating strong psychometric properties as evidenced by high reliability, validity, and treatment sensitivity [24].

### Data Analyses

#### Reliable Improvement and Recovery

Reliable improvement (evaluates whether a change in score is greater than the measurement error of the questionnaire) and recovery (scores changing from clinical to subclinical range) were calculated for the entire group as well as for the anxiety and depression subsamples. Individuals achieve reliable improvement when their GAD-7 score decreases by ≥4 points or their PHQ-9 score decreases by ≥6 points [25].

#### Growth Curve Modeling

Mixed effects modeling was used to examine the growth trajectories of the anxiety and depression scores over the course of the coaching sessions. The growth curve modeling approach allows incorporation of predictor variables at the response (PHQ-9, GAD-7) level and accounts for participant-level variability, obtaining average trajectories of the responses while acknowledging and accommodating differences/variability among participants. The results for each outcome are presented in a stepwise fashion, beginning with a null model containing only fixed and random effects corresponding to the growth function, followed by a series of conditional models incorporating response level predictors. All models featured a random effect on the intercept at the provider level as well as random effects for the intercept and linear effect of time (week) at the participant level.

## Results

### Reliable Improvement and Recovery

At baseline, 1404 (93.85%) of the 1496 clients scored in the clinical range on the GAD-7, and 216 clients (14.44%) scored in the clinical range on the PHQ-9. Table 2 reports the rates of reliable improvement and recovery based on the entire sample, as well as anxiety and depression subsamples.

**Table 2:** Reliable improvement and recovery rates. Clients achieved reliable improvement when the decrease in their GAD-7 score was ≥4 and/or the decrease in their PHQ-9 score was ≥6. Recovery only considers clients who started in the clinical range on the measure of interest.

Participant subgroup	Reliable improvement^a^, n (%)	Recovery^b^, n (%)	Reliable improvement and recovery^c^, n (%)	Reliable improvement or recovery^d^, n (%)
Entire group (N=1496)	1136 (75.94)	1326 (88.64)	1108 (74.06)	1339 (89.51)
GAD-7^e^ sample (n=1404)	1046 (74.50)	1229 (87.54)	1034 (73.65)	1241 (88.39)
PHQ-9^f^ sample (n=216)	148 (68.52)	202 (93.52)	148 (68.52)	202 (93.52)

^a^Calculated as reliable improvement in GAD-7 or PHQ-9 score.

^b^Calculated as recovery on the GAD-7 or PHQ-9.

^c^Calculated as reliable improvement and recovery on the GAD-7 *or* reliable improvement and recovery on the PHQ-9.

^d^Calculated as reliable improvement or recovery on the GAD-7 *or* reliable improvement or recovery on the PHQ-9.

^e^GAD-7: Generalized Anxiety Disorder-7 scale.

^f^PHQ-9: Patient Health Questionnaire-9.

### Growth Curve Modeling

A series of individual growth curve models were specified in the SAS PROC MIXED procedure (SAS Institute) and estimated using restricted maximum likelihood.

#### GAD-7 Results

Results from the unconditional analysis (Model 1) suggests that on average, participants exhibited a significant initial decline in GAD-7 during the first week of coaching (*b*=–1.27, 95% CI –1.32 to –1.22; *P*<.001), defined as 1 to 7 days after the initial session. Moreover, the presence of a significant quadratic coefficient (*b*=0.08, 95% CI 0.08 to 0.09; *P*<.001) indicates that the rate of decline in GAD-7 scores diminished over the course of coaching. More specifically, GAD-7 scores declined quickly over the first few weeks of coaching, although the average trajectory flattened gradually during the middle stages of coaching and more rapidly during the later stages.

In Model 2, significant coefficients emerged for coaching sessions (*b*=–0.90, 95% CI –1.03 to –0.78), which suggests that engaging in a coaching session was associated with a 0.90 decrease in the GAD-7 score during that same week (1 to 7 days after the session).

Model 3, the final model selected as representing the best fit for the data, incorporated the lagged engagement predictor, and a significant coefficient emerged for lagged coaching sessions (*b*=–0.74, 95% CI –0.87 to –0.61). This effect suggests that engaging in a coaching session was associated with 0.74 lower GAD-7 scores the following week (8 to 14 days after the coaching session). In this final model, all other coefficients from Model 1 (first week of coaching and quadratic coefficient) and Model 2 (coaching sessions in the past week) remained significant. Taken together, the clients exhibited a significant initial decline in anxiety symptoms during the first week of coaching as well as over the course of coaching, and engaging in a coaching session was associated with lower anxiety symptoms for the week immediately after that session and the following week. Results for each model are displayed in Tables 3 and 4.

**Table 3:** Growth curve modeling results on the Generalized Anxiety Disorder-7 scale (n=1404).

	Model 1		Model 2		Model 3	
	Estimate (95% CI)	*t* (observed)	Estimate (95% CI)	*t* (observed)	Estimate (95% CI)	*t* (observed)
Intercept	8.40 (8.29 to 8.51)	N/A^a^	8.68 (8.56 to 8.79)	N/A	8.87 (8.75 to 8.99)	N/A
Week	–1.27 (–1.32 to –1.22)	54.05^b^	–1.19 (–1.24 to –1.15)	50.21^b^	–1.03 (–1.08 to –0.97)	36.75^b^
Week^2	0.08 (0.08 to 0.09)	33.24^b^	0.07 (0.07 to 0.08)	29.53^b^	0.06 (0.05 to 0.06)	19.98^b^
Sessions, last 7 days	N/A	N/A	–0.90 (–1.03 to –0.78)	14.38^b^	–1.04 (–1.17 to –0.92)	16.45^b^
Sessions, 8-14 days	N/A	N/A	N/A	N/A	–0.74 (–0.87 to –0.61)	11.00^b^

^a^N/A: not applicable.

^b^*P*<.001.

**Table 4:** Model selection criteria results on the Generalized Anxiety Disorder-7 scale (n=1404).

Criterion	Model 1	Model 2	Model 3
Deviance (–2 log-likelihood)	32572.6	32373.7	32257.6
Akaike information criterion	32582.6	32383.7	32267.6
Bayesian information criterion	32600.3	32401.3	32285.2

#### PHQ-9 Results

Preliminary analyses on PHQ-9 scores revealed a relatively small degree of heterogeneity in patient level trajectories, sometimes resulting in a nonpositive definite covariance matrix of random effects at the patient level. However, sensitivity analyses revealed that this issue was resolved by constraining the intercept-slope covariance to 0. Several alternative specifications for patient-level random effects were also examined, though all configurations yielded the same conclusions for fixed-effect parameters. Results from the unconditional analysis (Model 1) revealed a steep initial decline in depression scores during the first week of coaching (*b*=–1.62, 95% CI –1.74 to –1.49; *P*<.001), and the significant quadratic coefficient (*b*=0.11, 95% CI 0.10 to 0.12; *P*<.001) indicates that the rate of decline in PHQ-9 scores diminished as coaching progressed. As seen in the anxiety analysis, depression scores declined rapidly over the first few weeks of coaching, but the average trajectory became flatter during the later stages.

In Model 2, significant coefficients emerged for coaching sessions (*b*=–0.62, 95% CI –0.94 to –0.30) indicating that engaging in a coaching session was associated with .62 lower PHQ-9 scores during that same week (1 to 7 days after the session).

Model 3, the final model selected as representing the best fit for the data, incorporated the lagged engagement predictor, and a significant coefficient emerged for lagged coaching sessions (*b*=–0.91, 95% CI –1.25 to –0.56). This effect suggests that each coaching session delivered was associated with 0.91 lower PHQ-9 scores in the following week (8-14 days after that coaching session). In this final model, all other coefficients from Model 1 (first week of coaching and quadratic coefficient) and Model 2 (coaching sessions in the past week, *b*=–0.79, 95% CI –1.11 to –0.46) remained significant. Taken together, clients exhibited a significant initial decline in depressive symptoms during the first week of coaching, as well as over the course of coaching, and engaging in a coaching session was associated with lower depressive symptoms for the week immediately after that session and the following week. Results for each model are displayed in Tables 5 and 6.

**Table 5:** Growth curve modeling results on the Patient Health Questionnaire-9 (n=216).

	Model 1			Model 2			Model 3	
	Estimate (95% CI)	*t* (observed)	Estimate (95% CI)		*t* (observed)	Estimate (95% CI)		*t* (observed)
Intercept	9.01 (8.68 to 9.34)	N/A^a^	9.20 (8.87 to 9.54)		N/A	9.43 (9.09 to 9.77)		N/A
Week	–1.62 (–1.74 to –1.49)	26.04^b^	–1.55 (–1.68 to –1.42)		24.07^b^	–1.34 (–1.49 to –1.19)		17.72^b^
Week^2	0.11 (0.10 to 0.12)	16.90^b^	0.10 (0.09 to 0.12)		15.12^b^	0.08 (0.07 to 0.10)		10.44^b^
Sessions, last 7 days	N/A	N/A	–0.62 (–0.94 to –0.30)		3.76^b^	–0.79 (–1.11 to –0.46)		4.76^b^
Sessions, 8-14 days	N/A	N/A	N/A		N/A	–0.91(–1.25 to –0.56)		5.15^b^

^a^N/A: not applicable.

^b^*P*<.001.

**Table 6:** Model selection criteria results on the Patient Health Questionnaire-9 (n=216).

Criterion	Model 1	Model 2	Model 3
Deviance (–2 log-likelihood)	5117.4	5105.3	5081.0
Akaike information criterion	5125.4	5113.3	5089.0
Bayesian information criterion	5136.7	5124.6	5100.3

## Discussion

Clients presenting with moderate levels of anxiety and depression exhibited a significant decrease in symptomology over the course of coaching, with close to 90% of clients with moderate anxiety and/or depression achieving reliable improvement or recovery. These findings suggest that cognitive-behavioral coaching is a promising method for managing these symptoms within a blended care context, assuming appropriate client selection. Additionally, it is noted that almost half of the sample belongs to an ethnic minority group. Achieving these strong outcomes within a diverse sample highlights the impact of providing culturally responsive care, as well as the potential for BCC to address some mental health disparities. Collectively, our findings are particularly exciting, as a substantial unmet need for mental health treatment remains in the United States given the shortage of licensed mental health providers.

When clients presented for coaching sessions, significant decreases in anxiety and depressive symptoms were observed for that same week as well as for the following week, reflecting the importance of session attendance. These findings highlight the crucial role of the coach in introducing and reinforcing the clinical concepts and skills in each session, which is an integral part of the blended care model. Coaches are also able to personalize the digital activities and place them in the context of the clients’ presenting issues, enhancing the precision of care. Additionally, the sessions are valuable opportunities to troubleshoot emergent issues and mitigate issues with homework compliance. Given that between-session homework compliance is a significant driver of symptom change [26], the increased accountability afforded by the coaching sessions is imperative for optimal outcomes. Taken together, coaching sessions allow for personalization of care and enhance engagement with clinical concepts and skills, contributing to durable decreases in anxiety and depression symptoms.

Additionally, the steepest declines in symptoms tended to occur during the initial weeks of coaching, emphasizing the importance of client buy-in and early engagement. The beginning stages of coaching are crucial for establishing the foundational rationale for the work to come. Solid psychoeducation about the purpose of a BCC model and how it can alleviate anxiety and depression is imperative for establishing positive expectations. Indeed, more positive expectations have been linked to better outcomes [27], highlighting the importance of collaborative care and dedicating time to clarify goals and expectations at the outset [28]. Establishing this initial momentum will also highlight the results of clients’ efforts firsthand, which is expected to maintain their buy-in and motivation to continue their work in BCC.

These findings should be considered within the context of certain limitations and future directions. First, over half of individuals seeking care were never offered the BCC program due to more severe clinical presentations such as suicidality, which limits the generalizability of the findings. Although it is a limitation to generalizability, the careful consideration that was given in determining who would be appropriate for this modality of care (eg, clients with moderate or lower levels of anxiety and depression) is arguably a strength of this program, as it is not suggested that coaching should be a substitute to therapy across a range of clinical severity. Second, the clients included in our analysis most commonly presented with anxiety and depression; future studies should evaluate the effectiveness of a BCC program for other psychiatric issues. Third, coaches in this program received extensive training in cognitive behavioral concepts, whereas traditional coaching focuses more narrowly on motivational interviewing and goal setting. Fourth, as coaching session outcomes were determined via client self-reports, it could be informative to incorporate multimethod (eg, clinical interview), multi-informant (eg, reports from significant others/family members) assessments in future evaluations for a more comprehensive understanding of symptomology and functioning. Finally, although there were robust reductions of anxiety and depression, findings were derived from a naturalistic study that examined retrospective data, which limits our ability to account for regression to the mean effects. As such, it would be helpful to conduct randomized controlled trials to impart further confidence in the effectiveness of BCC programs in reducing these symptoms beyond a control condition. Future dismantling studies should also be conducted to determine which components of the BCC model contributed to the biggest change in symptom severity. Engagement with digital CBT content (independent of coaching) has been shown to yield positive symptom improvement. By teasing apart the unique contributions of the digital components and the face-to-face coaching sessions, the active ingredients for change can be identified and fortified.

Ultimately, these data provide strong support for the use of an evidence-based CBC program within a blended care model for clients presenting with moderate levels of anxiety and depression. Under appropriate clinical supervision to ensure proper client selection and oversight, coaching appears to be a promising modality to expand access to timely mental health treatment.

## Abbreviations

ACT: acceptance and commitment therapy
BCC: blended care coaching
CBC: cognitive-behavioral coaching
CBT: cognitive behavioral therapy
DBT: dialectical behavior therapy
GAD-7: Generalized Anxiety Disorder-7 scale
HIPAA: Health Insurance Portability and Accountability Act
PHQ-9: Patient Health Questionnaire-9
